# Azacitidine might be beneficial in a subgroup of older AML patients compared to intensive chemotherapy: a single centre retrospective study of 227 consecutive patients

**DOI:** 10.1186/1756-8722-6-29

**Published:** 2013-04-16

**Authors:** Lieke H van der Helm, Ellen RM Scheepers, Nic JGM Veeger, Simon MGJ Daenen, André B Mulder, Eva van den Berg, Edo Vellenga, Gerwin Huls

**Affiliations:** 1 Department of Haematology, University Medical Centre Groningen, University of Groningen, Hanzeplein 1, Groningen, 9713 GZ, The Netherlands; 2 Department of Laboratory Medicine, University Medical Centre Groningen, University of Groningen, Groningen, The Netherlands; 3 Department of Genetics, University Medical Centre Groningen, University of Groningen, Groningen, The Netherlands

**Keywords:** Acute myeloid leukaemia, Older age, Azacitidine, Intensive chemotherapy, Best supportive care

## Abstract

**Background:**

Treatment options in older acute myeloid leukaemia (AML) patients include intensive chemotherapy, best supportive care (BSC), and hypomethylating agents. Currently, limited data is available on hypomethylating agents in older AML patients in unselected patient populations.

**Methods:**

To compare the effectiveness of azacitidine with conventional therapy, we collected data of 227 consecutive AML patients (≥60 years) who were treated with azacitidine (*N* = 26), intensive chemotherapy (*N* = 90), or BSC (*N* = 97).

**Results:**

Azacitidine-treated patients were older and had more comorbidities, but lower white blood cell- and bone marrow blast counts compared with intensive chemotherapy patients. Complete or partial response was achieved in 42% of azacitidine-treated patients and in 73% of intensive chemotherapy patients (*P* = 0.005). However, the overall survival (OS) was similar (1-year-OS 57% versus 56%, *P* = 0.93; 2-year-OS 35% versus 35%, *P* = 0.92), and remained similar after correction for risk factors in a multivariate analysis. Patients treated with BSC had an inferior OS (1-year- and 2-year-OS 16% and 2%, *P <* 0.001). Compared to intensive chemotherapy, azacitidine-treated patients spent less days in the hospital (median in first three months 0.5 versus 56, *P* < 0.001), and needed less red blood cell and platelet transfusions (median per month 2.7 versus 7, *P* < 0.001 and 0.3 versus 5, *P* < 0.001) in the first three months.

**Conclusions:**

Azacitidine treatment is associated with a comparable OS but higher tolerability in a subgroup of older AML patients compared with intensive chemotherapy. Patients receiving BSC had a poor prognosis.

## Background

Acute myeloid leukaemia (AML) is characterised by a differentiation defect of haematopoietic stem- and progenitor cells, leading to the accumulation of blast cells and cytopenias. The incidence of AML increases with age, with a median age at diagnosis of approximately 70 years [1]. Older AML patients generally have a poor prognosis compared to younger patients due to a higher incidence of comorbidities, higher rates of treatment related mortality, and adverse disease characteristics, associated with resistant disease and relapses [2-5]. Median overall survival (OS) of patients over 60 years of age treated with intensive chemotherapy is less than 1 year, with complete remission (CR) rates of about 50% and a treatment related mortality of at least 15%, indicating an unfavourable risk-benefit ratio of intensive chemotherapy [6,7].

Nevertheless, several studies suggest that older AML patients benefit from treatment. A small randomized clinical trial by the HOVON study group and an analysis of the Swedish Acute Leukemia Registry showed that standard intensive treatment improves early death rates and long-term survival compared to best supportive care only (BSC) in older patients [8-10]. In an additional prospective randomized trial it was demonstrated that low-dose cytarabine treatment was superior to BSC and hydroxyurea in patients with favourable- or intermediate-risk cytogenetics [11].

The optimal treatment of older AML patients in daily clinical practice remains challenging. A choice should be made between intensive chemotherapy, less intensive treatment, and palliation, considering individual risks and benefits [7,12]. To guide physicians in their decisions, several prognostic factors have been identified and risk scores have been developed based on age, performance status, comorbidities, cytogenetics, molecular markers, clinical variables, and laboratory measurements [13-17].

Recently, the DNA methyltransferase inhibitor azacitidine has become available for MDS and AML patients with up to 30% bone marrow blast. A superior OS has been demonstrated in AML patients with 20–30% bone marrow blasts treated with azacitidine compared to conventional treatment [18,19]. Two recent studies showed a beneficial outcome in previously untreated AML patients, including patients with more than 30% bone marrow blasts, who were treated with azacitidine [20,21]. However, limited data is available on the treatment of older unselected AML patients with azacitidine compared to conventional treatment options. To study the impact of azacitidine and conventional care options in routine clinical practice, we analysed the treatment results of 227 consecutive newly diagnosed AML patients of 60 years and older in our centre.

## Results

### Baseline characteristics of the study population

The study population included 227 consecutive newly diagnosed AML patients of 60 years and older. Patients were treated according to one of the following main strategies: 26 (11%) patients were treated with azacitidine, 90 (40%) patients were treated with intensive chemotherapy, 97 (43%) patients were treated with best supportive care (BSC), and 14 (6%) patients were diagnosed with acute promyelocytic leukaemia (APL) and treated with all-trans retinoic acid (ATRA)-containing intensive chemotherapy (Additional file 1: Figure S1). These fourteen APL patients were excluded from comparisons of azacitidine with intensive chemotherapy, because of the alternative treatment strategy and superior OS. Patients who were treated with azacitidine completed a median of 6 (1–30) cycles. All azacitidine patients started with the standard dose (7 days 75 mg/m^2^/day), except for one patient with pancytopenia and malaise at diagnosis who received 50 mg/m^2^/day. A dose reduction of 30% was made in one patient after 10 cycles and a schedule change from 7 to 5 days was applied in one patient after 13 cycles. Failure to receive at least three cycles of azacitidine was reported in six (23%) patients; the reason of interruption was early death (*N =* 4), or pancytopenia (*N =* 2).

Baseline patient- and disease characteristics of the different treatment groups are shown in Table 1. Considering patient related factors: patients who were treated with azacitidine and BSC were significantly older than patients treated with intensive chemotherapy (*P* < 0.001). Patients receiving azacitidine had a superior performance (*P* = 0.002), and patients receiving BSC had a worse performance (*P* = 0.003) compared to patients receiving intensive chemotherapy. The HCT-comorbidity score was worse in patients treated with azacitidine (*P* = 0.029) and BSC (*P* < 0.001) compared to intensive chemotherapy. Considering disease related factors: the percentage of secondary AML (including therapy related AML, prior MDS, and prior myeloproliferative neoplasms) was not significantly different between the treatment groups (*P =* 0.089). Patients who were treated with azacitidine had lower bone marrow (BM) blast counts (*P* < 0.001), and white blood cell (WBC) counts (*P* < 0.001) compared to patients treated with intensive chemotherapy. Indeed, none of the patients treated with azacitidine had WBC ≥ 15 × 10^9^/l. The cytogenetic risk score was not significantly different in patients treated with azacitidine compared with intensive chemotherapy (*P* = 0.48), but cytogenetic risk was worse in patients treated with BSC compared to intensive chemotherapy (*P* = 0.005). In 23 patients, the karyotype was not evaluated at baseline.

**Table 1:** Baseline patient- and disease characteristics by treatment strategy

	**All patients (*N *=213)**	**Azacitidine (*N *=26)**	**Intensive chemotherapy (*N *=90)**	**BSC (*N *=97)**	***P*-value**	
					**Overall**	**Aza vs IC**
**Age**						
Median (range)	68 (60–96)	70 (60–81)	66 (60–74)	71 (60–96)	<0.001	<0.001
≥ 70 years	80 (38%)	14 (54%)	10 (11%)	56 (58%)	<0.001	<0.001
**Sex**					0.48	0.27
Male	119 (56%)	17 (65%)	47 (52%)	55 (57%)		
**Performance score**					<0.001	0.002
≥ 2	121 (59%)	5 (19%)	47 (54%)	69 (75%)		
**HCT-comorbidity index**					<0.001	0.029
Low (0)	98 (46%)	9 (35%)	57 (63%)	32 (33%)		
Intermediate (1–2)	66 (31%)	8 (31%)	18 (20%)	40 (41%)		
High (> 2)	49 (23%)	9 (35%)	15 (17%)	25 (26%)		
**AML FAB classification**					0.27	0.28
M0/M1	41 (20%)	4 (17%)	21 (21%)	16 (19%)		
M2	87 (42%)	8 (33%)	40 (40%)	39 (47%)		
M4/M5	51 (25%)	11 (46%)	22 (22%)	18 (22%)		
M6/M7	13 (6%)	1 (4%)	4 (4%)	8 (10%)		
**AML type**					0.089	0.056
*De novo*	139 (65%)	13 (50%)	65 (72%)	61 (63%)		
Secondary	74 (35%)	13 (50%)	25 (28%)	36 (37%)		
**Bone marrow blasts**						
Median (range)	45 (16–100)	27 (20–88)	52 (20–100)	47 (16–93)	<0.001	<0.001
≥ 30%	135 (70%)	11 (42%)	67 (77%)	57 (72%)	0.003	0.001
**WBC**						
Median (range)	5 (0–360)	3 (0–15)	5 (1–236)	7 (1–360)	0.13	<0.001
≥ 15 × 10^9^/l	65 (31%)	0 (0%)	31 (34%)	34 (35%)	0.002	<0.001
**LDH**						
Median (range)	324 (116–4835)	259 (136–1133)	340 (134–2664)	332 (116–4835)	0.15	0.092
> 600 U/l	49 (23%)	2 (8%)	21 (23%)	26 (27%)	0.13	0.15
**Cytogenetic risk**					0.003	0.48
Favourable	8 (4%)	0 (0%)	4 (4%)	4 (4%)		
Intermediate	135 (63%)	18 (69%)	62 (69%)	55 (57%)		
Unfavourable	47 (22%)	8 (31%)	21 (23%)	18 (19%)		
Not available	23 (11%)	0 (0%)	3 (3%)	20 (21%)		
**Molecular markers**					0.27	0.45
*NPMc+/ITD-*	13 (7%)	1 (4%)	9 (11%)	3 (4%)		
Others	163 (93%)	23 (96%)	75 (89%)	65 (96%)		

Patients with promyelocytic leukaemia (*N* = 14) were excluded from this analysis. Abbreviations: BSC, best supportive care only; Aza vs IC, azacitidine versus intensive chemotherapy; HCT, hematopoietic cell transplantation; AML, acute myeloid leukaemia; BM, bone marrow; WBC, white blood cell count; LDH, lactate dehydrogenase; *NPMc+/ITD-*, cytoplasmic *NPM1* without *FLT3* internal tandem duplication. Results are reported as *N* (%) unless otherwise indicated.

Allogeneic hematopoietic stem cell transplantation (allo-SCT) was applied in 14 patients following induction chemotherapy and also in one patient after azacitidine treatment. Baseline characteristics of these allo-SCT patients are shown in Additional file 2: Table S1. All allo-SCT patients received reduced intensity conditioning with fludarabine (30 mg/m^2^ for 3 subsequent days) and 2 Gray total body irradiation (TBI) before transplantation. All patients received mobilised peripheral blood stem cells, that were obtained from HLA matched siblings in 11 patients and from matched unrelated donors in 4 patients. Since transplant strategies have evolved during the study period, the last years, patients under the age of 70 in CR after two cycles of chemotherapy with a 10/10 matched donor available received an allogeneic hematopoietic cell transplantation. Indeed, of the patients younger than 70 years in CR, 12 (24%) received an allo-SCT, which is 10 (48%) considering this patient group since 2008.

### Response

Response (CR, PR) was achieved in 11 (42%) patients who were treated with azacitidine, and in 66 (73%) patients who were treated with intensive chemotherapy, which was significantly different (*P* < 0.001; Table 2). Of the 55 patients treated with 6-mercaptopurine, two patients met criteria for PR. In the azacitidine group, median time to response from the start of therapy was 4 months (range 3–7 months) and median duration of response was 16 months. Of the 15 azacitidine-treated patients who did not meet criteria for response, 5 patients had a stable disease for 5–15 months. In the intensive chemotherapy group, CR was achieved in 46 (51%) patients after the first induction cycle and, cumulatively, in 58 (64%) patients after the second induction cycle. Median duration of response in the intensive chemotherapy group was 11 months.

**Table 2:** Treatment outcome of patients treated with azacitidine or intensive chemotherapy

	**Azacitidine (*N *=26)**	**Intensive chemotherapy (*N *=90)**		***P*-value**	
		**All (*N *=90)**	**Excl. allo-SCT (*N =*** **76)**	**Aza vs all IC**	**Aza vs IC excl. allo-SCT**
**Overall survival**					
1-year	57%	56%	50%	0.93^1^	0.80^1^
2-year	35%	35%	31%	0.92^1^	0.50^1^
**Response, overall**	**11 (42%)**	**68 (76%)**	**54 (71%)**	<0.001	0.005
CR	9 (35%)	63 (70%)	49 (65%)		
PR	2 (8%)	5 (6%)	5 (7%)		
No CR or PR	15 (58%)	22 (24%)	22 (29%)		
**Early death**					
within 4 weeks	1 (4%)	4 (4%)	4 (4%)	0.88^1^	0.79^1^
within 8 weeks	2 (8%)	11 (12%)	11 (12%)	0.51^1^	0.40^1^
**Relapse/death after response**					
within 1 year	4 (36%)	39 (57%)	34 (63%)	0.21^1^	0.18^1^
within 2 years	5 (45%)	42 (62%)	37 (69%)	0.30^1^	0.14^1^
**Days in hospital,** median (range)					
month 1–3	0.5 (0–30)	56 (2–85)	54 (2–85)	<0.001	0.029
month 4–6	0 (0–8)	0 (0–81)	0 (0–81)	0.036	0.006
**RBC transfusions,** median per month (range)					
month 1–3	2.7 (0–10)	7 (0–32)	7 (0–32)	<0.001	<0.001
month 4–6	0 (0–13)	1 (0–8)	0 (0–8)	0.97	0.65
**PLT transfusions,** median per month (range)					
month 1–3	0.3 (0–7)	5 (0–19)	5 (0–19)	<0.001	<0.001
month 4–6	0 (0–1)	0 (0–8)	0 (0–8)	0.016	0.047

^1^Log rank test. Patients with promyelocytic leukaemia (*N* = 14) were excluded from this analysis. Abbreviations: excl. allo-SCT, excluding patients undergoing allogeneic hematopoietic stem cell transplantation; IC, intensive chemotherapy; Aza, azacitidine; vs, versus; CI, confidence interval; CR, complete remission; PR, partial remission; RBC, red blood cell; PLT, platelet. Results are reported as *N* (%) unless otherwise indicated.

### Early mortality and supportive care

The 4- and 8-week mortality rates and the relapse rates were not significantly different in the azacitidine group compared with the intensive chemotherapy group. However, the number of days in the hospital was significantly lower in patients treated with azacitidine compared to intensive chemotherapy during the first three months (0.5 versus 56 days, *P <* 0.001) and the following 3 months, i.e. months 4–6 (0 (range 0–8) versus 0 (range 0–81) days, *P =* 0.036) after diagnosis (Table 2; Additional file 3: Figure S2A). Patients treated with azacitidine needed less red blood cell transfusions (2.7 versus 7, *P* < 0.001) and less platelet transfusions (0.3 versus 5, *P* < 0.001) during the first three months after diagnosis compared to patients treated with intensive chemotherapy, but the number of red blood cell transfusions during months 4–6 was similar in both treatment groups (0 versus 0.7, *P* = 0.97) (Table 2; Additional file 3: Figure S2 B, C). Similar results were obtained when excluding patients who underwent allo-SCT (Table 2).

### Complications and causes of death

To compare the number of complications and the causes of death, we selected patients who were treated in the time period that azacitidine was used (2009–2012). Grade 3/4 infections occurred in 9 (35%) azacitidine-treated patients, in 9 (32%) BSC-treated patients, and in all 46 (98%, 1 missing) patients who received intensive chemotherapy. Of the patients treated with azacitidine, 17 (65%) had grade 3/4 anaemia or thrombocytopenia at some time during the treatment. Causes of death in patients treated with azacitidine, BSC, or intensive chemotherapy were disease progression in 8 (62%), 19 (79%), and 12 (50%) patients, respectively; infection in combination with progressive disease in 4 (31%), 5 (21%), and 4 (17%) patients, respectively; and infection without disease progression in 0 (0%) azacitdine and BSC patients, but in 7 (30%) intensive chemotherapy patients. One patient treated with intensive chemotherapy died because of ischemic heart disease. The patient who was treated with azacitidine and allo-SCT died because of graft-versus-host disease.

### Impact of patient- and disease related factors on overall survival

Median OS of all 227 patients was 7.8 months. Patients with a good performance score (0–1) at baseline had a better OS than patients with an adverse score (≥2) (12.6 versus 4.0 months, respectively; *P* < 0.001) (Additional file 4: Figure S3A and Additional file 5: Table S2). Cytogenetic risk significantly predicted the survival with a median OS of 5.9 months in patients with favourable-risk cytogenetics, excluding APL patients, 9.7 months in patients with intermediate risk cytogenetics, and 3.6 months in patients with unfavourable-risk cytogenetics (*P* < 0.001) (Additional file 4: Figure S3B and Additional file 5: Table S2). Patients with a translocation t(15;17) (APL) had a superior OS (median not reached) compared to other AML patients. The median OS of patients with no karyotype available was 1.9 months, which was similar to the OS of patients with unfavourable-risk cytogenetics (*P* = 0.33) (Additional file 4: Figure S3B and Additional file 5: Table S2). The nucleophosmin 1 (*NPM1*) mutation status and presence of *FLT3*-internal tandem duplication (ITD) were determined in 67 of the 80 patients with a normal karyotype. Nine patients had cytoplasmic *NPM1* without *FLT3-ITD* (*NPMc+/ITD-*). Although numbers are small, a trend towards better OS was observed in these patients compared to patients without *NPMc+/ITD-* (median OS 29.5 versus 8.5 months; *P* = 0.12; Additional file 4: Figure S3C).

### Impact of treatment on overall survival

**Figure 1:**
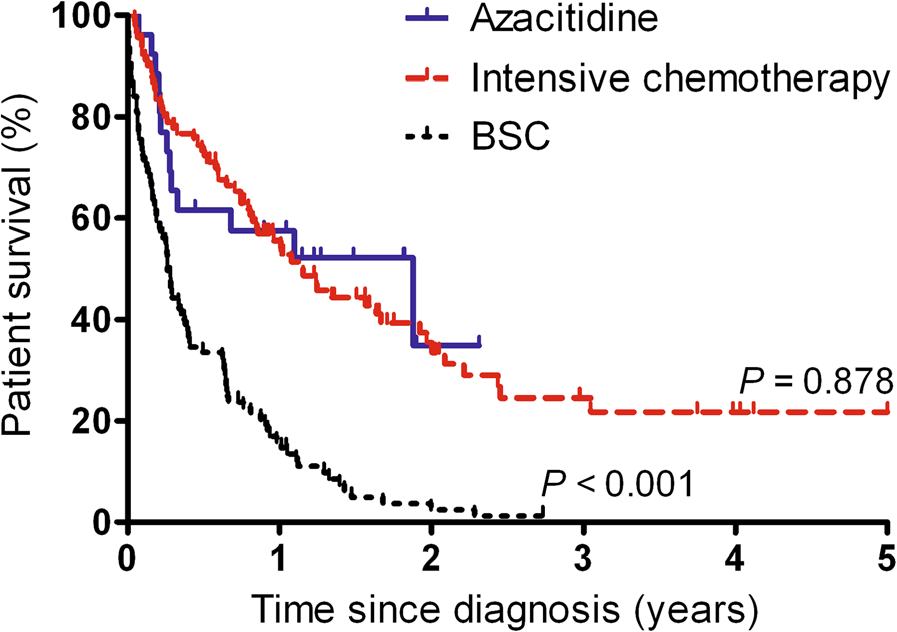
**Overall survival by treatment strategy.** The OS is similar in patients who were treated with azacitidine (*N* = 26) and intensive chemotherapy (IC; *N* = 90), and is worse in patients who received BSC (*N* = 97).

The OS in the different treatment groups is depicted in Figure 1. The OS was similar in patients receiving azacitidine and patients receiving intensive chemotherapy (Table 2; 1-year OS 57% versus 56%, *P* = 0.93; 2-year OS 35% versus 35%, *P* = 0.92). Also when we compared the OS of patients treated since 2009, when azacitidine became available, we observed a similar OS in patients receiving azacitidine and patients receiving intensive chemotherapy (Additional file 6: Figure S4; 1-year OS 57% versus 51%, *P* = 0.80; 2-year OS 35% versus 38%, *P* = 1.00). Since baseline differences were present among patients treated with azacitidine versus intensive chemotherapy, we assessed the OS in the subgroups of patients aged ≥70 years, patients with HCT comorbidity score >0, patients with performance scores <2, patients with <30% BM blasts, and patients with <15 × 10^9^/l WBC’s. Also in these subgroups, no significant differences in OS were observed between azacitidine and intensive chemotherapy (*P =* 0.74*; P =* 0.71*; P =* 0.25*; P =* 0.71*; P =* 0.95*,* respectively).

Patients who received BSC had a significantly worse OS (1-year and 2-year OS 16% and 2%) compared to azacitidine and intensive chemotherapy (*P* < 0.001 and *P* < 0.001). When we selected for patients with a good performance score (<2), which was the lesser part of the BSC group, we still observed a significantly worse OS in the BSC group compared to azacitidine (*P =* 0.025) and intensive chemotherapy (*P =* 0.004).

The median OS of fifteen patients who underwent allo-SCT was 22.5 months from the date of diagnosis, while other patients treated with intensive chemotherapy or azacitidine had a median OS of 12.9 months (p = 0.05). After allo-SCT, five patients died due to a relapse (*N* = 4) or graft versus host disease (*N =* 1).

Of the 90 patients treated with intensive chemotherapy, 51 (57%) were included in a clinical trial and 39 (43%) were treated off-study. Patients treated off-study had more comorbidities (p < 0.001) than patients included in a trial. Between these patient groups, no differences in overall response rates (75% versus 72%; *P* = 0.81), and no differences in median OS (14.9 versus 12.9 months, respectively; *P* = 0.77) were observed (data not shown).

### Predictors for overall survival

To assess whether the OS was similar in patients who were treated with azacitidine and intensive chemotherapy after correction for patient- and disease related factors, we performed a multivariate regression analysis. First, we determined which factors were associated with OS. In univariate analysis, unfavourable OS was associated with BSC (versus azacitidine), unfavourable cytogenetic risk or cytogenetic risk not evaluated (versus intermediate risk), age ≥70 years, performance score ≥2, and LDH >600 U/l (Additional file 5: Table S2). Next, we selected from univariate analysis predictors for OS with *P* < 0.10. Multivariate analysis confirmed BSC, unfavourable cytogenetic risk, and LDH >600 U/l as independent adverse predictors for OS. The survival of patients treated with azacitidine versus intensive chemotherapy was not significantly different after correction for these factors (P = 0.84).

## Discussion

In this single centre retrospective study, treatment results of 227 newly diagnosed consecutive AML patients aged ≥60 years who have been treated with BSC, azacitidine, or intensive chemotherapy, were analysed. This study confirms the dismal prognosis of older AML patients who receive only BSC, which was either related to adverse characteristics at baseline or to the treatment type. To optimise treatment in older patients who are unfit for chemotherapy, new therapies are developed, including azacitidine. A treatment benefit for azacitidine compared to BSC was observed in a post-hoc analysis of the AML patients in the AZA-001 randomized trial [19]. In the same trial, also a limited number of patients treated with intensive chemotherapy was included, but no significant differences in OS were observed between patients treated with azacitidine versus intensive chemotherapy.

In our retrospective study, despite the limitations of the relatively small number of patients and disparities between the treatment groups, we observed no significant differences in OS in patients treated with azacitidine compared to intensive chemotherapy. Also a time-dependent effect could be excluded. When corrected for baseline differences in a multivariate analysis, a HR of 1.07 was found with a 95% CI of 0.58–2.0 when azacitidine and intensive chemotherapy were compared. Despite relatively small numbers resulting in a wide CI, our point estimate (HR = 1.07) does suggest a comparable treatment effect of azacitidine treatment versus intensive chemotherapy in older AML patients with good performance scores and low WBC counts. Comparable results have been reported recently by the MD Andersen Cancer Centre in a cohort study of 671 patients, including 114 patients treated with hypomethylation-based (either azacitidine or decitabine) therapy [22]. In this study they also reported a significant difference in CR rates but similar OS in patients treated with epigenetic therapy versus intensive chemotherapy. These observations, in a larger cohort, are in line with our observations and might suggest that the currently used response criteria are not sufficient for evaluating some (less intensive) treatment strategies. Further, in the perspective of comparing intensive treatment with less intensive treatment, it is also interesting to note that a small prospective randomised trial between chemotherapy and low-dose cytarabine did not result in a survival benefit for intensive treatment [23].

An important issue, though difficult to analyse, is the reason why some patients received only BSC, others azacitidine and others intensive chemotherapy. The patients receiving azacitidine differed from the intensive chemotherapy patients in terms of older age, and more comorbidities, but also better performance, lower WBC counts, and lower BM blast counts, while the BSC group consisted of older patients with a high cytogenetic risk score, a poor performance score, and a high HCT-comorbidity index. Apparently, although no defined guidelines were used, the treating physicians seem to have integrated these baseline characteristics in their clinical decisions. Azacitidine is currently only registered for the treatment of AML with bone marrow blasts between 20% and 30%. However, we have recently analysed a cohort of 55 AML patients treated in different hospitals with azacitidine, which included 31% patients with ≥30% bone marrow blasts. A comparable OS and response rates were demonstrated in patients with <30% and ≥30% bone marrow blasts (van der Helm, 2013, in publication). These findings are in line with the results of the Italian named patient program and a German trial [20,21]. An additional advantage of azacitidine is the tolerability [19,24,25], which is reflected in our study by a lower number of days in the hospital and a lower number of red blood cell- and platelet transfusions compared to intensive chemotherapy. In addition, only two of 26 azacitidine-treated patients discontinued treatment because of drug toxicity.

The ongoing phase III trial of azacitidine versus BSC versus intensive chemotherapy (AZA-AML-001 trial) is expected to finally provide the decisive answers for the optimal treatment schedule for elderly AML patients. Recently, the results have been reported of a large phase III trial, comparing the efficacy and safety of decitabine (20 mg/m^2^, days 1–5) (*N* = 242) with treatment choice (supportive care (*N* = 28) and low dose cytarabine (*N* = 215) of older patients with newly diagnosed AML and poor- or intermediate-risk cytogenetics [26]. The authors concluded that there was a significant improvement in median OS with decitabine versus treatment choice.

## Conclusions

Azacitidine treatment is associated with a comparable OS but higher tolerability in a subgroup of older AML patients compared with intensive chemotherapy. Patients receiving BSC had a poor prognosis. Therefore, our data suggest that azacitidine treatment might be a valuable alternative to intensive chemotherapy and should be considered instead of BSC in older AML patients.

## Methods

### Patients and data collection

For this retrospective study, data has been collected from 227 consecutive AML patients of 60 years and older who were diagnosed and treated between January 2002 and May 2012 at the University Medical Centre Groningen. Patients were entered in the study after approval by a scientific review committee and the University Medical Center Groningen Institutional Review Board. Informed consent was obtained in accordance with the Declaration of Helsinki. Data has been collected by studying health records of individual patients between October 2011 and October 2012. The minimal follow-up time was six months. Diagnoses were made using French-American-British criteria and World Health Organization (WHO)-2008 criteria [27,28]. Cytogenetic risk was defined according to the National Comprehensive Cancer Network (NCCN) guidelines [29]. Baseline comorbidity was quantified by the haematopoietic cell transplantation (HCT) comorbidity index, which was previously demonstrated to be a predictive score in AML patients over 60 years of age treated with intensive chemotherapy [14,30,31].

### Treatment

Azacitidine was available in The Netherlands from December 2008 onwards in a compassionate named patient program. Azacitidine was administered subcutaneously at the approved schedule of 75 mg/m^2^/day during 7 days every 28 days. It was intended to give at least 6 cycles of azacitidine and to continue treatment until progression in patients who responded well. Dose reductions and delays of treatment cycles could be made.

Intensive chemotherapy was administered according to one of the HOVON studies [9,32,33], which all contain standard dose cytarabine and an anthracycline (http://www.hovon.nl). As part of the subsequent HOVON studies, patients were randomised to receive or not G-CSF, intermediate dose cytarabine, bevacizumab, clofarabine, or lenalidomide in addition to the chemotherapy. Of the patients treated according to HOVON studies, 57% was officially included in a HOVON study. Allogeneic haematopoietic stem cell transplantation could be applied following induction therapy. Patients with acute promyelocytic leukaemia (APL) were treated with ATRA-containing chemotherapy, according to the HOVON 79 study [34].

Best supportive care (BSC) consisted of transfusions, antibiotics, and hospital admissions as needed. 6-Mercaptopurine and hydroxycarbamide could be added to the treatment. Red blood cell- or platelet transfusions were given in agreement with general recommendations: Hb <8 g/dl, or higher in case of comorbidity, and platelets <20 × 10^9^/l or higher in case of bleeding or anticoagulant therapy.

### Response criteria and study endpoints

Response was evaluated after every treatment cycle of intensive chemotherapy and azacitidine by blood count and by bone marrow aspirate if available. Morphologic CR and partial remission (PR) were defined according to IWG-2003 criteria for AML [35]. Response duration was measured from the date at which marrow evaluation took place in patients achieving CR or PR, until relapse or death or censoring. OS was measured from the date of diagnosis. Patients who remained alive were censored at the time of the last visit to the hospital.

### Statistical analysis

Differences between groups in patient characteristics and response rates were compared using 2-sided Fisher’s exact tests or chi-square tests for categorical variables and Kruskal-Wallis tests or Wilcoxon tests for quantitative variables, unless otherwise indicated. Survival curves were estimated with the Kaplan-Meier method and differences in survival were calculated by logrank tests. Predictive factors for OS were analysed by Wald tests for univariate and multivariate comparisons. For multivariate analysis, we selected variables with *P* < 0.10 in univariate analysis. Cox proportional hazards regression models were used to estimate hazard ratios (HR) and associated 95% confidence intervals (CI). As we aimed to compare azacitidine treatment with intensive chemotherapy and BSC, the variable “treatment strategy” was pre-added to the multivariate regression model. A *P*-value <0.05 was considered significant. SPSS-20 was used for analysis.

## Findings

Response rates were lower in older AML patients treated with azacitidine compared to intensive chemotherapy, however, overall survival in these treatment groups was comparable.

## Abbreviations

Allo-SCT: Allogeneic haematopoietic stem cell transplantation; AML: Acute myeloid leukaemia; APL: Acute promyelocytic leukaemia; ATRA: All-trans retinoic acid; BM: Bone marrow; BSC: Best supportive care; CI: Confidence interval; CR: Complete remission; HCT: Haematopoietic cell transplantation; HR: Hazard ratio; ITD: Internal tandem duplication; LDH: Lactate dehydrogenase; NPM1: Nucleophosmin 1; OS: Overall survival; PR: Partial remission; RBC: Red blood cell transfusion; PLT: Platelet transfusion; WBC: White blood cell count; WHO: World Health Organization.

## Competing interests

The authors declare that they have no competing interests.

## Authors’ contributions

LHvdH and ERMS provided the conception and design of the study, acquisition of data, analysis and interpretation of data, drafting the manuscript, revised it critically for important intellectual content. NJGMV supplied the analysis and interpretation of data. SMGJD enrolled patients and provided acquisition of data. ABM and EvdB provided acquisition of data. EV and GH provided the conception and design of the study, interpretation of data, drafting the manuscript, revised it critically for important intellectual content. All authors read and approved the final manuscript.

## Supplementary Material

Additional file 1: Figure S1 Flow diagram of the study population. Between January 2002 and May 2012, 227 consecutive AML patients aged ≥60 years were diagnosed and treated in our hospital. Abbreviations: AML, acute myeloid leukaemia; APL, acute promyelocytic leukaemia. Click here for file

Additional file 2: Table S1 Baseline characteristics of patients who underwent allogeneic haematopoietic stem cell transplantation. Click here for file

Additional file 3: Figure S2 Supportive care during treatment with azacitidine or intensive chemotherapy. (A) The number of days in the hospital was lower in patients treated with azacitidine compared to intensive chemotherapy during the first three months (*P* < 0.001) and the following 3 months (*P =* 0.036) after diagnosis. (B) Patients treated with azacitidine needed less platelet (PLT) transfusions during the first three months (*P* < 0.001) and the following three months (*P* < 0.016) compared to intensive chemotherapy. (C) Patients treated with azacitidine needed less red blood cell (RBC) transfusions (*P <* 0.001) during the first three months compared to intensive chemotherapy. The median, 5^th^, 25^th^, 75^th^, and 95^th^ percentile are depicted. Click here for file

Additional file 4: Figure S3 Impact of patient and disease factors on overall survival. (A) Patients with WHO performance score 0–1 had a superior OS compared to patients with performance score ≥2. (B) The cytogenetic risk score was a strong predictor for OS. Patients with acute promyelocytic leukaemia (APL) had a favourable survival. The OS of patients with no cytogenetics available was comparable to patients with unfavourable-risk cytogenetics. (C) In patients with a normal karyotype, a trend towards better OS was observed in the presence of cytoplasmic *NPM1* without *FLT3-ITD* (*NPMc+/ITD-*) compared to other patients. Click here for file

Additional file 5: Table S2 Predictors for overall survival: univariate and multivariate analysis. Click here for file

Additional file 6: Figure S4 Overall survival by treatment strategy in the time period that azacitidine was available. The OS was similar in patients who were treated with azacitidine (*N* = 26) and intensive chemotherapy (IC; *N* = 47), and was worse in patients who received BSC (*N* = 28). Click here for file
