# Supplementary figures and images for: Azacitidine might be beneficial in a subgroup of older AML patients compared to intensive chemotherapy: a single centre retrospective study of 227 consecutive patients
Source: J Hematol Oncol. 2013 Apr 16;6:29. doi: 10.1186/1756-8722-6-29 (PMC3639930; doi:10.1186/1756-8722-6-29)

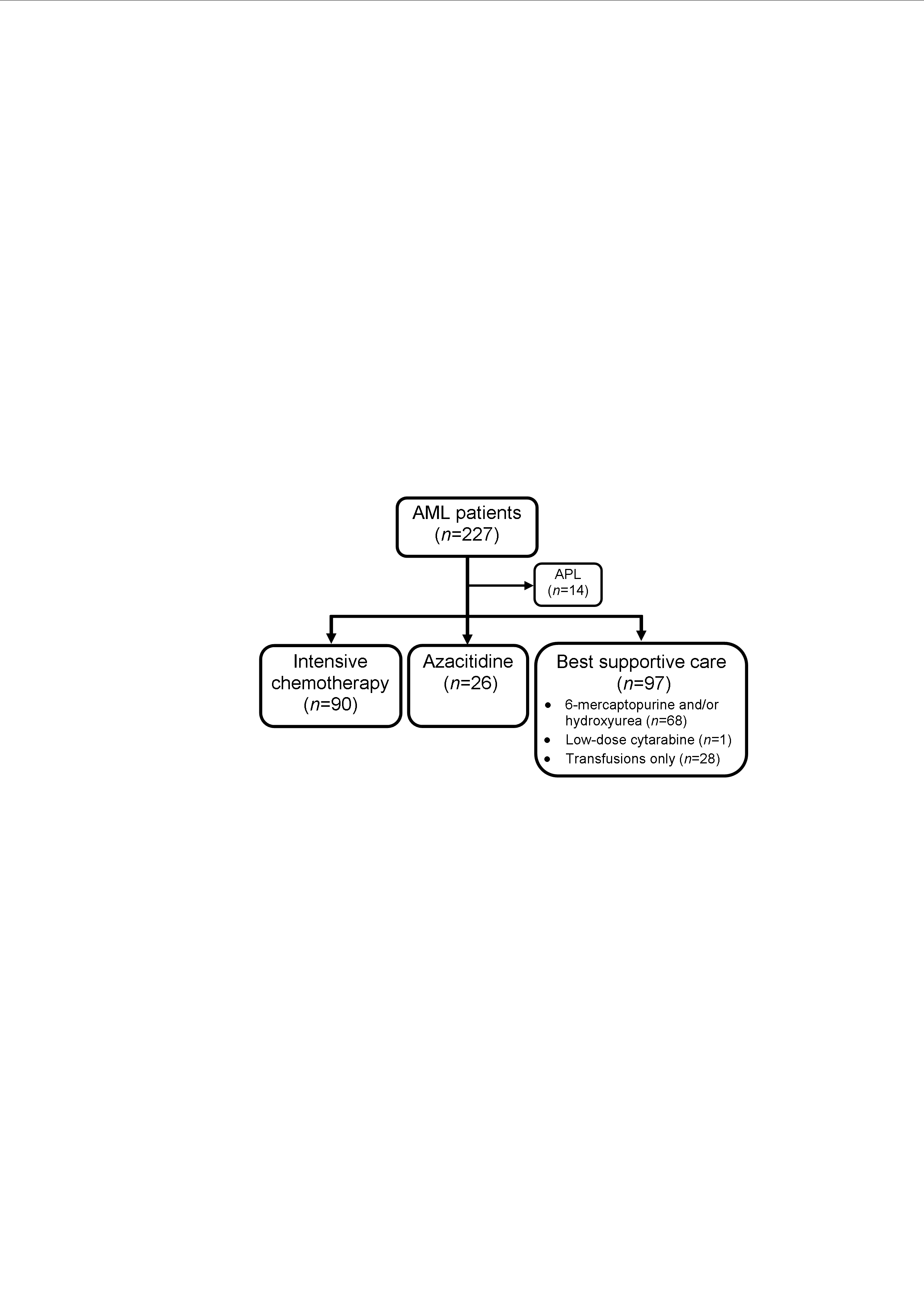

Supplement: Additional file 1: Figure S1 — Flow diagram of the study population. Between January 2002 and May 2012, 227 consecutive AML patients aged ≥60 years were diagnosed and treated in our hospital. Abbreviations: AML, acute myeloid leukaemia; APL, acute promyelocytic leukaemia. [file 1756-8722-6-29-S1.tiff]

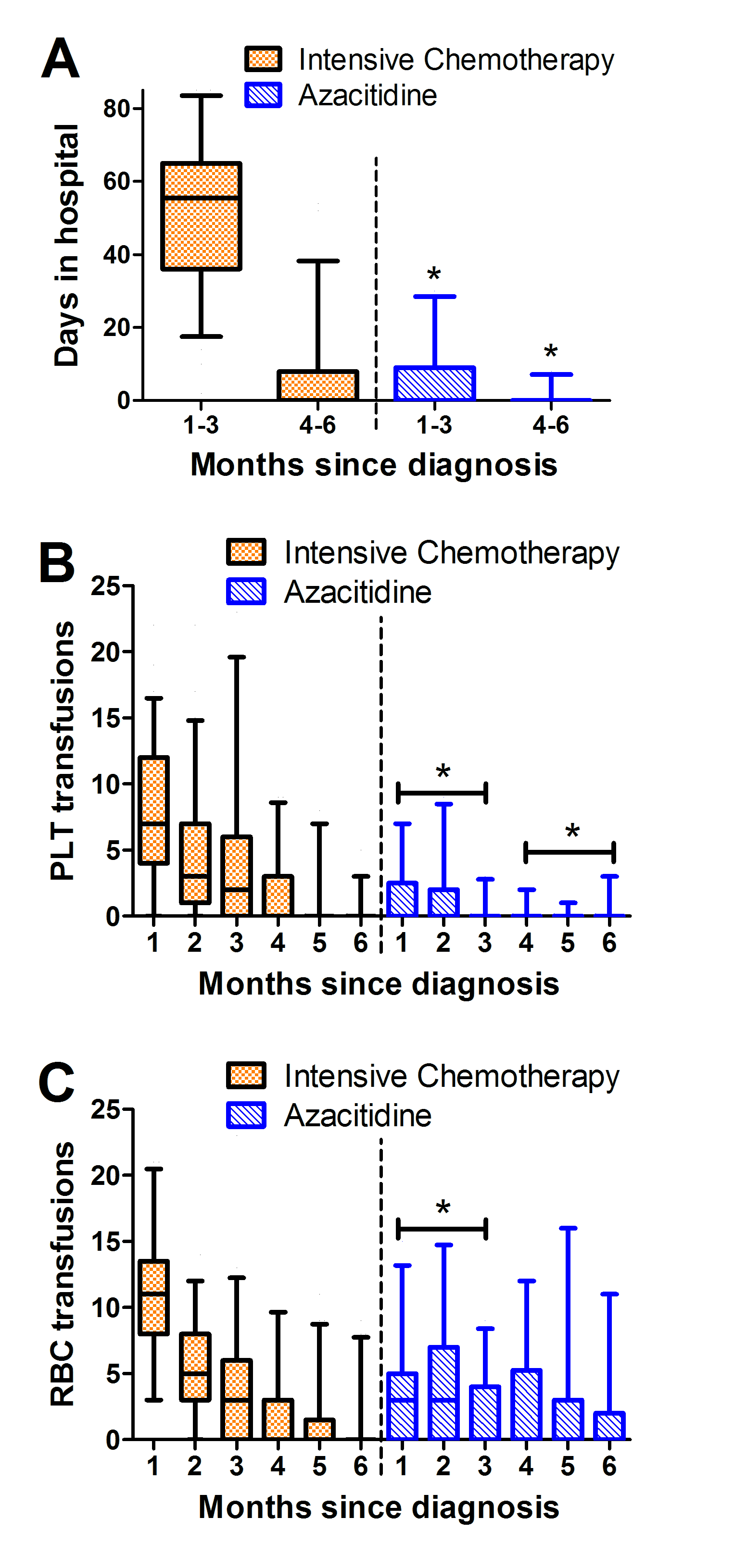

Supplement: Additional file 3: Figure S2 — Supportive care during treatment with azacitidine or intensive chemotherapy. (A) The number of days in the hospital was lower in patients treated with azacitidine compared to intensive chemotherapy during the first three months (P < 0.001) and the following 3 months (P = 0.036) after diagnosis. (B) Patients treated with azacitidine needed less platelet (PLT) transfusions during the first three months (P < 0.001) and the following three months (P < 0.016) compared to intensive chemotherapy. (C) Patients treated with azacitidine needed less red blood cell (RBC) transfusions (P < 0.001) during the first three months compared to intensive chemotherapy. The median, 5th, 25th, 75th, and 95th percentile are depicted. [file 1756-8722-6-29-S3.tiff]

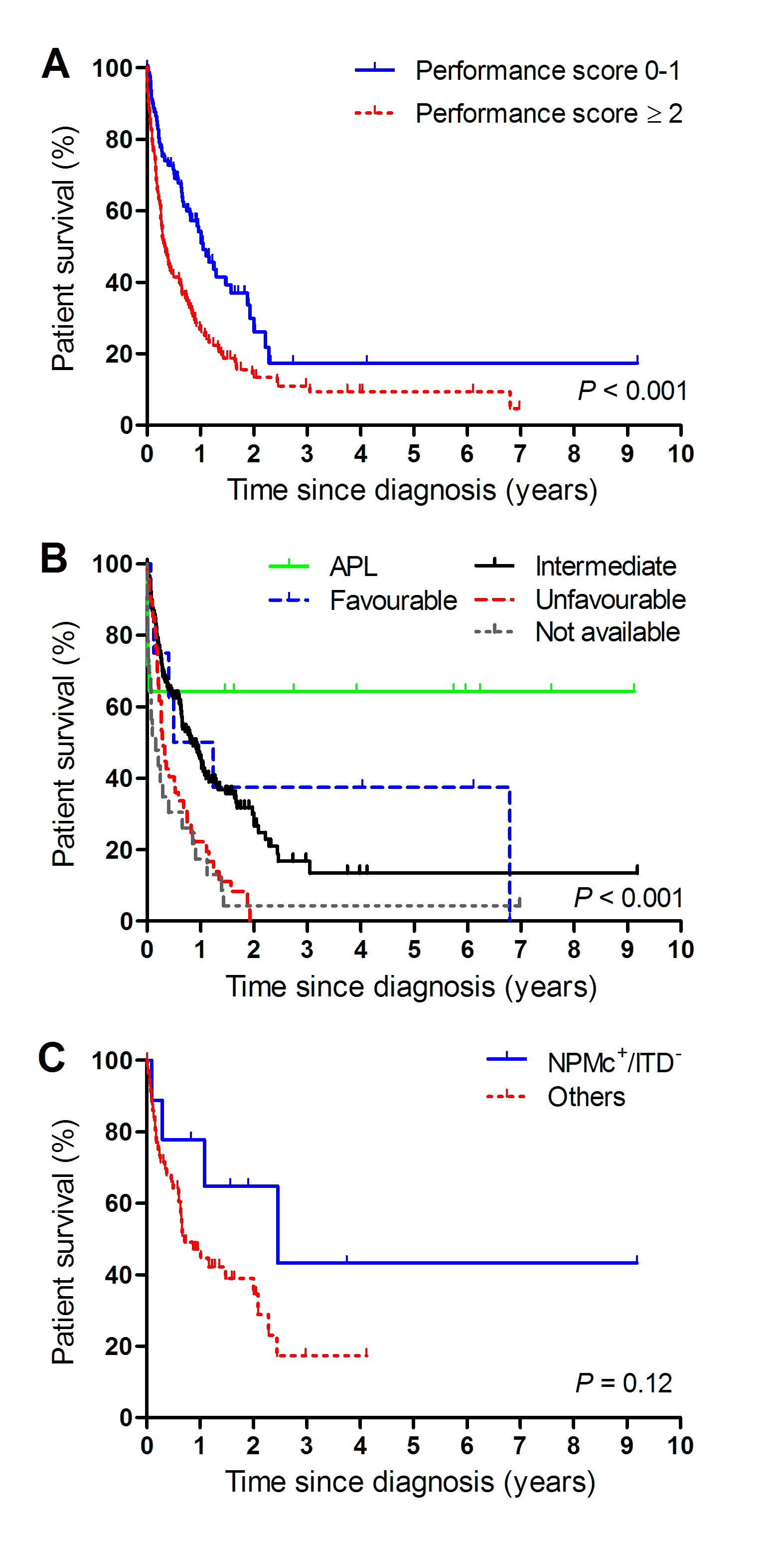

Supplement: Additional file 4: Figure S3 — Impact of patient and disease factors on overall survival. (A) Patients with WHO performance score 0–1 had a superior OS compared to patients with performance score ≥2. (B) The cytogenetic risk score was a strong predictor for OS. Patients with acute promyelocytic leukaemia (APL) had a favourable survival. The OS of patients with no cytogenetics available was comparable to patients with unfavourable-risk cytogenetics. (C) In patients with a normal karyotype, a trend towards better OS was observed in the presence of cytoplasmic NPM1 without FLT3-ITD (NPMc+/ITD-) compared to other patients. [file 1756-8722-6-29-S4.tiff]

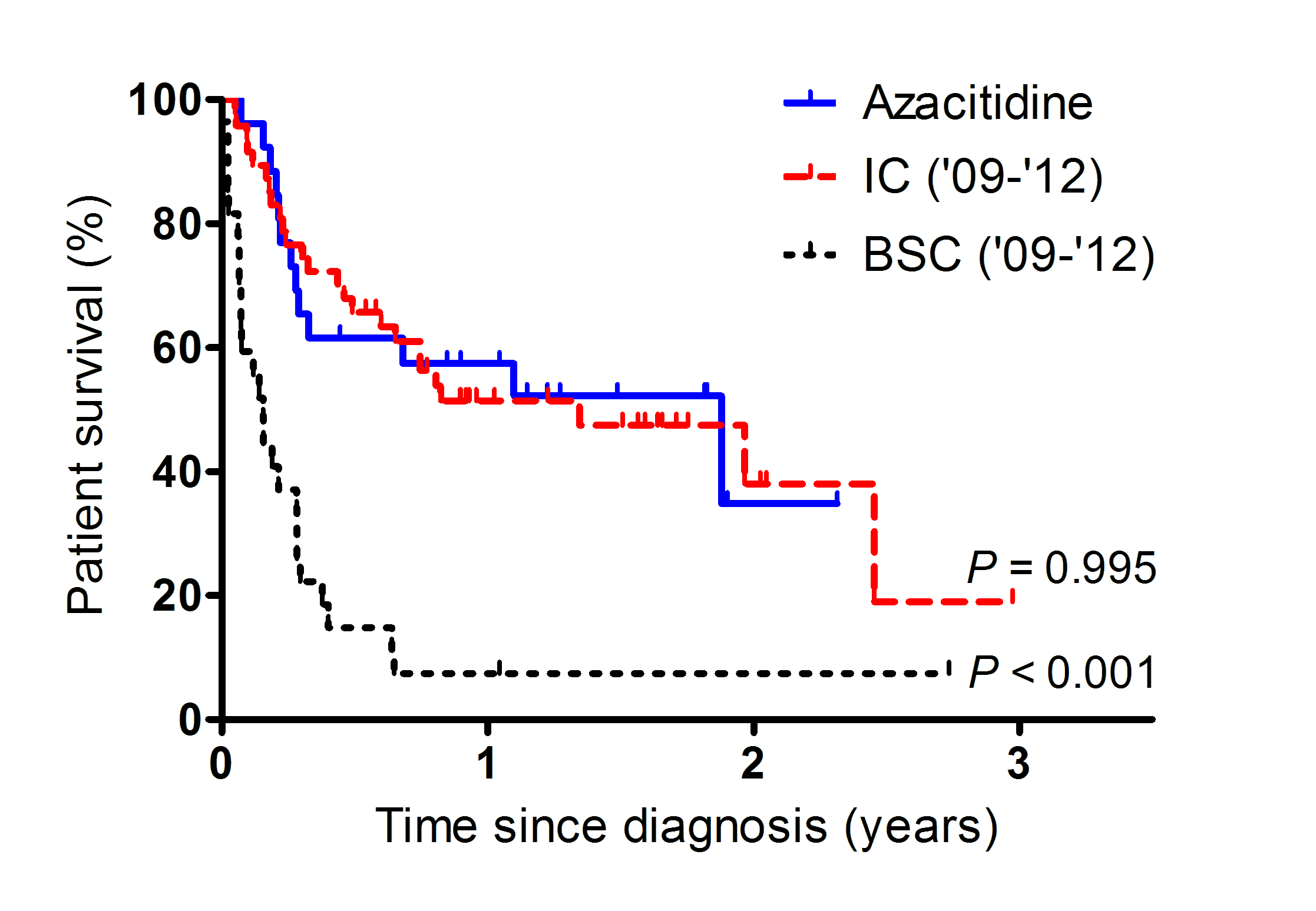

Supplement: Additional file 6: Figure S4 — Overall survival by treatment strategy in the time period that azacitidine was available. The OS was similar in patients who were treated with azacitidine (N = 26) and intensive chemotherapy (IC; N = 47), and was worse in patients who received BSC (N = 28). [file 1756-8722-6-29-S6.tiff]
