# Supplementary material for: Azacitidine might be beneficial in a subgroup of older AML patients compared to intensive chemotherapy: a single centre retrospective study of 227 consecutive patients
Source: J Hematol Oncol. 2013 Apr 16;6:29. doi: 10.1186/1756-8722-6-29 (PMC3639930; doi:10.1186/1756-8722-6-29)
Supplement: Additional file 2: Table S1 — Baseline characteristics of patients who underwent allogeneic haematopoietic stem cell transplantation. [file 1756-8722-6-29-S2.doc]

**Table S1. Baseline characteristics of patients who underwent allogeneic hematopoietic stem cell transplantation**

|  | Allo-SCT patients  (*N* =14) |
| --- | --- |
|
| **Age,** median (range) | 64 (60-68) |
| **Sex,** male | 9 (60%) |
| **Performance score,** ≥ 2 | 5 (33%) |
| **HCT-comorbidity index**  Low (0)  Intermediate (1-2)  High (> 2) | 11 (73%)  4 (27%)  0 (0%) |
| **AML FAB classification**  M0/M1  M2  M4/M5  M6/M7 | 2 (13%)  8 (53%)  4 (27%)  1 (7%) |
| **AML type**  *De novo*  Therapy related  Prior MDS/myeloproliferative disease | 9 (60%)  2 (13%)  4 (27%) |
| **Bone marrow blasts,** median (range)  ≥ 30% | 47 (21-69)  10 (67%) |
| **WBC,** median (range)  ≥ 15 x10^9^/l | 4 (1-31)  4 (27%) |
| **LDH,** median (range)  > 600 U/l | 253 (134-1224)  4 (27%) |
| **Cytogenetic risk**  Favourable  Intermediate  Unfavourable | 0 (0%)  11 (73%)  4 (27%) |
| **Molecular markers**  *NPMc+/ITD-*  Other | 0 (0%)  15 (100%) |
