# Supplementary material for: Azacitidine might be beneficial in a subgroup of older AML patients compared to intensive chemotherapy: a single centre retrospective study of 227 consecutive patients
Source: J Hematol Oncol. 2013 Apr 16;6:29. doi: 10.1186/1756-8722-6-29 (PMC3639930; doi:10.1186/1756-8722-6-29)
Supplement: Additional file 5: Table S2 — Predictors for overall survival: univariate and multivariate analysis. [file 1756-8722-6-29-S5.doc]

**Table S2. Predictors for overall survival: univariate and multivariate analysis**

|  | Median OS (months) | HR (95% CI) | *P*-value |
| --- | --- | --- | --- |
| 1. Univariate analysis |  |  |  |
| **Treatment strategy** |  |  | <0.001 |
| Intensive chemotherapy | 13.8 | Ref. |  |
| Azacitidine | 22.5 | 0.95 (0.52-1.7) | 0.87 |
| BSC | 3.1 | Ref. |  |
| Azacitidine | 22.5 | 0.30 (0.17-0.53) | <0.001 |
| Intensive chemotherapy | 13.8 | Ref. |  |
| BSC | 3.1 | 3.2 (2.3-4.5) | <0.001 |
| **Cytogenetic risk** |  |  | <0.001 |
| Favourable | 5.9 | 0.78 (0.34-1.8) | 0.56 |
| Intermediate | 9.7 | Ref. |  |
| Unfavourable | 3.6 | 2.0 (1.4-2.9) | <0.001 |
| Not available | 1.9 | 2.4 (1.5-3.8) | <0.001 |
| **AML FAB classification** |  |  | 0.77 |
| M0/M1 | 10.3 | Ref. |  |
| M2 | 18.8 | 1.0 (0.68-1.6) | 0.85 |
| M4/M5 | 13.8 | 0.94 (0.57-1.5) | 0.80 |
| M6/M7 | 20.0 | 1.4 (0.68-2.7) | 0.38 |
| **AML type** |  |  |  |
| *De novo* | 7.9 | Ref. |  |
| Secondary | 4.8 | 1.3 (0.91-1.7) | 0.16 |
| **Age** |  |  |  |
| < 70 years | 9.5 | Ref. |  |
| ≥ 70 years | 3.9 | 1.5 (1.1-2.1) | 0.007 |
| **Performance score** |  |  |  |
| 0-1 | 12.6 | Ref. |  |
| ≥ 2 | 4.0 | 1.8 (1.3-2.5) | <0.001 |
| **WBC** |  |  |  |
| < 15 x10^9^/l | 8.0 | Ref. |  |
| ≥ 15 x10^9^/l | 4.8 | 1.4 (0.98-1.9) | 0.068 |
| **Bone marrow blasts** |  |  |  |
| < 30% | 13.4 | Ref. |  |
| ≥ 30% | 7.5 | 1.4 (0.94-1.9) | 0.10 |
| **LDH** |  |  |  |
| ≤600 U/l | 8.1 | Ref. |  |
| >600 U/l | 3.4 | 1.6 (1.1-2.3) | 0.012 |
| 1. Multivariate analysis | | | |
| **Treatment strategy** |  |  | <0.001 |
| Intensive chemotherapy | 13.8 | Ref. |  |
| Azacitidine | 22.5 | 1.07 (0.58-2.0) | 0.84 |
| BSC | 3.1 | Ref. |  |
| Azacitidine | 22.5 | 0.32 (0.18-0.59) | <0.001 |
| Intensive chemotherapy | 13.8 | Ref. |  |
| BSC | 3.1 | 3.3 (2.3-4.7) | <0.001 |
| **Cytogenetic risk** |  |  | <0.001 |
| Favourable | 5.9 | 0.85 (0.36-2.0) | 0.71 |
| Intermediate | 9.7 | Ref. |  |
| Unfavourable | 3.6 | 2.4 (1.6-3.5) | <0.001 |
| Not available | 1.9 | 1.5 (0.89-2.4) | 0.14 |
| **LDH** |  |  |  |
| ≤600 U/l | 8.1 | Ref. |  |
| >600 U/l | 3.4 | 1.8 (1.2-2.6) | 0.002 |

Patients with promyelocytic leukaemia (*N*=14) are excluded from this analysis. Abbreviations: OS, overall survival; HR, hazard ratio; CI, confidence interval; Ref., reference group; BSC, best supportive care only; AML, acute myeloid leukaemia; WBC, white blood cell count; LDH, lactate dehydrogenase.
